# Estimating the value of face coverings during the COVID-19 epidemic: a dynamic causal modelling study

**DOI:** 10.1136/bmjph-2025-003489

**Published:** 2025-12-25

**Authors:** Cam Bowie, Anthony Costello, Karl Friston

**Affiliations:** 1 Somerset Health Authority (retired), Axminster, UK; 2 Institute for Global Health, University College London, London, UK; 3 Queen Square Institute of Neurology, University College London, London, UK

**Keywords:** COVID-19, Epidemiology, Public Health, Communicable Disease Control, statistics and numerical data

## Abstract

**Introduction:**

The value of face coverings in the pandemic has been disputed. Dynamic causal modelling (DCM) allows one to test hypotheses about the nature of viral spread and effectiveness of interventions. Our aim was to quantify the impact of face coverings on viral transmission and deaths associated with COVID-19 between 2020 and 2023 in the UK.

**Methods:**

A DCM of the COVID-19 outbreak in the UK was used. It combines conventional (ie, Susceptible Exposed Infectious Recovered (SEIR)) epidemiological and sociobehavioural models to simultaneously fit measures of prevalence, clinical data, vaccine use and fluctuations in mobility. The model was augmented to include an effect of reported use of face coverings within the past week by adults in reducing community transmission, which was then suppressed to see what might have happened in their absence.

**Results:**

We found face coverings by adults reduced COVID-19 transmission by 5% in adults (90% CI 4.6% to 5.6%) and the elderly (90% CI 4.8% to 5.6%), but only by 2.3% in children (90% CI 1.5% to 3.4%) and had negligible effects in younger adults (0.4%, 90% CI 0.2% to 0.8%). This effect on transmission had a large impact on mortality, with an estimated saving of 102 000 (90% CI range 40 000 to 164 000) lives over the 30-month study period, suggesting a 50% reduction in mortality.

**Conclusions:**

Modelling the impact of widely used face coverings during the pandemic revealed a quantitatively small (age-dependent) impact on community transmission that translates into a marked public health impact, as estimated by the prevention of deaths.

**WHAT IS ALREADY KNOWN ON THIS TOPIC**

- There is a dispute about the value of face coverings in viral epidemics. Quality meta-analysis of randomised trials suggests a risk ratio of 0.86 for face coverings in community settings in PCR positive COVID-19 cases, but which is not significant.

**WHAT THIS STUDY ADDS**

- Applying dynamic causal modelling to timeseries data accumulated during the COVID-19 epidemic finds that face coverings are effective in reducing community transmission in the UK during the first 30 months of the epidemic by 5% in adults. Crucially, the credible intervals (90% CI 4.6 to 5.6) provide very strong evidence for an effect of face coverings (that lies comfortably within the CIs based on randomised controlled trials).

**HOW THIS STUDY MIGHT AFFECT RESEARCH, PRACTICE OR POLICY**

- The importance of this modelling lies in its application—to the population of the UK—with the implication that a national policy of face covering had a substantive effect in reducing disease and death. This effect can be quantified using scenario modelling based on the above estimates, demonstrating a disproportionately large effect on COVID-19-related deaths (ie, face covering prevented an excess mortality of about 50%).

## Introduction

 The value of face coverings in the pandemic has been disputed; a recent multidisciplinary review considered the results of modelling exercises that assessed the value of face coverings and found them unhelpful.^1^ The authors argued for more sophisticated modelling. The dynamic causal model (DCM) approach is well suited to assessing the value of face coverings, both in terms of their effect on transmission and their effect on reducing infectiousness and population death rates.

**Figure 1:**
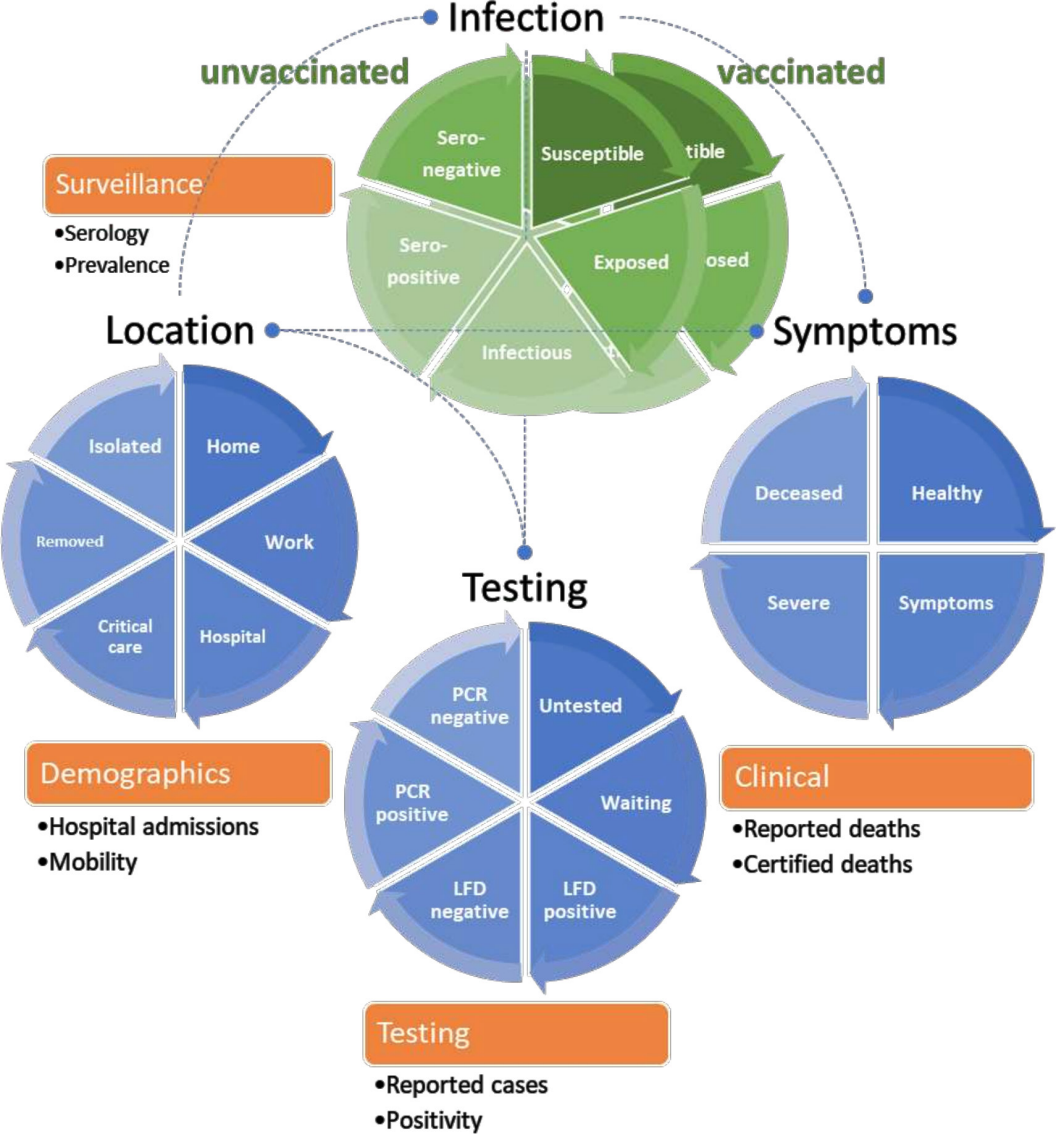
Dynamic causal model of COVID-19.This schematic summarises the form of the DCM used for the current analyses (5). The circles represent four latent factors, while the segments correspond to the latent states of each factor. The green factor is the closest to a conventional epidemiological (eg, SEIR) model. This (infection) factor is augmented within sociobehavioural (location and testing) and clinical (symptoms) factors. The states within any factor are mutually exclusive. In other words, every individual in the population has to be in one state or another. The orange boxes represent the observable data that are generated by this model. The rate of transition between states—or the dwell time within any state—rests upon the model parameters that, in some instances, can be specified with fairly precise priors. The broken lines indicate that transitions among states—within a factor—depend on the occupancy of states in another factor. For example, the probability of moving from a susceptible state of infection to exposed (ie., infected but not infectious) depends upon location, which affects contact rate, and so on. The model includes a *seronegative* state to model individuals who have acquired (e.g., T-cell) immunity following infection or via cross-reactivity^6 7^ or other protective host factors.^8 9^ Three states of the infection factor are duplicated to model vaccination. This allows one to parameterise differential susceptibility to infection and pathogenicity when vaccinated—and a return to unvaccinated state—to model waning immunity. Similarly, all four factors were duplicated to model four age groups, with parameterised contact rates among the ensuing groups (0-14 years; 15-34 years; 35-69 years and 70 years and older). Please see for details and technical references.^3^ LFD, lateral flow device.

DCM is an efficient and expressive form of complex system modelling that eschews stochastic simulations by using variational procedures to model population density dynamics generating time-series data.^2^,^5^ DCM has the particular advantage of furnishing a measure of model evidence (ie, variational free energy) that is more reliable than usual estimators, such as the Akaike information criterion and Bayesian information criterion. This is important because it allows one to test hypotheses about the nature of viral spread—or effectiveness of interventions—by comparing models with and without a particular effect.^2^ The application of DCM is outlined in table 1 using the DCM for COVID-19 to illustrate the procedures. The structure of the DCM for COVID-19 is summarised in figure 1 in terms of the various factors and states that generate outcomes or data. These data are used to estimate the most likely model and its parameters.

**Table 1:** Dynamic causal modelling

	Dynamic causal modelling	The COVID-19 model
1	Define the system Identify the (hidden) states of the system generating (observable) data Identify all possible causal links among states and how states cause data	A compartmental model with four factors The COVID-19 model is a compartmental population model in which (hidden or latent) states are divided into four factors, such that any individual—in the population—has to be in one of that factor’s states. The factors here include the location of an individual, their state of infection, their symptomatic state and testing status (see figure 1).
2	Formulate models Parameterise the causal links in a way that allows one to test hypotheses by removing parameters	This paper compares two models The full or parent model includes a parameter mediating the putative effect of face covering on transmission risk. By removing or suppressing this parameter we create a reduced model in which face covering has no effect
3	Gather data Collect and organise all the available time-series data the model was designed to generate.	40 kinds of timeseries The timeseries data used here are supplied by the ONS, UKHSA and Google and report recorded cases, deaths, vaccine, testing, travel, retail activity, transport data and so on. Some of these timeseries are age-stratified. Some are dense (eg, daily); others are sparse (eg, monthly)
4	Fit models to data Use variational Bayes to estimate the posterior probability density over model parameters, given the data	Parameter estimation using variational Laplace 61 parameters are calculated which best fit the data. How? An approximate posterior density is optimised to maximise a variational bound on the logarithm of model evidence or marginal likelihood using standard (gradient descent) procedures. Effectively, this updates a prior density over parameters into a posterior density, after seeing the data. See online supplemental table 1 for a list of the (61) parameters and their densities
5	Compare models Use the posterior to evaluate model evidence and compare reduced models to test hypotheses or remove redundant parameters	Bayesian model reduction to assess the effectiveness of face coverings Here, we use Bayesian model reduction to test the hypothesis that face coverings had an effect on transmission risk. If we suppress the parameter mediating this effect, we can see whether model evidence goes up or down. If it goes down, there is evidence for an effect of face coverings. If it goes up, it means that the face covering parameter is redundant and the original (full) model was too complex
6	Infer causality Report the model parameters (and credible intervals) mediating causal influences	Report the posterior inferences about the face covering parameter Here, we ensure independence of the face covering parameter from other parameters. How? We plot the posterior probability density over the face covering parameter for each age group and then report the posterior correlations with other parameters. See figure 3.
7	Interpret results Explain the effects of changing or removing parameters in terms of their effects on outcomes	Illustrate the consequences of face coverings on mortality Here, we generate data using the posterior estimates of the parameters before and after reducing the face covering parameter. This furnishes a form of scenario modelling, enabling one to interpret the effect of face coverings in terms of their consequences. See figure 4.

* An overview using the COVID-19 model as an example.

ONS, Office of National Statistics; UKHSA, UK Health Security Agency.

The DCM for COVID-19 was used during the pandemic to provide long-term forecasts—on a fortnightly basis—from February 2021 to November 2023.^6 7^ Published reports using this DCM have examined the epidemic in different countries and regions, various mitigation strategies and the reliability of projections of the model in peer-reviewed journals and numerous preprints.^2^,^68^ For the purposes of the current analyses, the model was equipped with an extra parameter modelling the effect of face coverings on transmission risk, to enable a retrospective estimation of their effectiveness and subsequent scenario modelling (ie, with and without face covering) to evaluate the contribution of face covering to public health.

## Materials and methods

### The dynamic causal modelling

DCM offers two complementary approaches to understanding the use of face coverings. One can ask why people wear face coverings—by treating empirical measures of the propensity to wear face coverings as an observable consequence of unobservable (latent) states—that is, ask questions about what *causes* people to wear a face covering, such as the risk of getting infected. Alternatively, one can treat observed face covering as a cause of latent states that generate data—that is, ask questions about the effects of face coverings.

In this report, we focus on the effectiveness of face covering, modelled as a potential reduction in transmission risk. DCM for COVID-19 is a particularly apt model to ask questions about this because it is one of the most expressive and realistic models of viral spread available. We used the version of DCM for COVID-19 that was last used for forecasting in November 2023 (and remains available from the UCL website). This DCM generalises conventional (eg, SEIR) epidemiological models by adding clinical and sociobehavioural factors to fit multiple data modalities; ranging from the usual measures of prevalence, morbidity and mortality, through vaccine use and testing, to fluctuations in economic activity and mobility. The predictive validity of the DCM, used in this report, has been established in a series of publications, suggesting it is better in terms of its predictive accuracy—sometimes by an order of magnitude—than alternative modelling initiatives.^8 10 11^ This model validation means that incorporating the effect of face covering calls for a minimal change to the model structure that is sufficient for answering the question: did face covering make a difference?

The model was, therefore, equipped with an extra parameter based on the following logic: the probability that I will become infected, given I am susceptible, depends on (1) the probability that you—the person I meet—is infected (ie, prevalence), (2) the probability that I will encounter you (ie, contact rate) and (3) the probability that you will transmit the virus to me, if we are in close contact (ie, transmission risk). The effect of face covering was therefore modelled as a reduction in transmission risk. There are several determinants of transmission risk. The DCM already accommodates variations in transmissibility due to the emergence of new variants and seasonality effects. To include face covering, we supplemented the seasonality effects S(*t*) ∈ [0, 1], with an effect due to the propensity to wear face coverings, M(*t*) ∈ [0, 1]—as functions of time in days—with the following parameterisation of transmission risk:


$$P(t)=erf(P_{v}.P_{u})\in [0,1]$$

$$P_{v}=\sum_{\tau }\theta_{\tau }{\;}^{tra.}(erf((t-\Delta_{\tau })/32)+1)/2§gt;0$$

$$P_{u}=\theta^{trn}+\theta^{trm.}S(t)-\theta^{msk.}M(t)§gt;0$$

The error function (*erf*) is a sigmoid function—returning values between minus one and plus one—that passes through the origin. This parameterisation ensures that transmission risk varies between zero and one. *P_v_* corresponds to increases in transmissibility with the emergence of new variants parameterised by θ_t_^tra^. The (*erf*) basis functions modelling increases in transmissibility at ∆_t_ were specified by the points of inflection, as one variant overtook another:

4 November 2020 for Alpha.

10 May 2021 for Alpha to Delta.

10 December 2021 for Alpha to Omicron BA.1.

14 February 2022 for Omicron BA.1 to Omicron BA.2.

6 June 2022 for Omicron BA.2 to Omicron BA.5.

These dates correspond to the points of inflection, at which point one variant overtook another. The emergence of successive variants was therefore modelled as smooth increases in transmission risk over a month (ie, 32 days), as one variant replaced another. The time constant of 32 days was based on the serological tracking of successive variants.

To model the time-dependent effects of seasonality and face coverings P_u_, we added an extra parameter, θ^msk^ that scaled the contribution of face covering M(*t*) **∈** [0,1]. Although there is a small increase in self-reported face covering of adults with age, we elected to use the same face covering function for all age groups, based on non-age stratified data. For example, average use of a face covering (from 21 May 2020 to 27 March 2022) was 84.9% in people aged 16–29 years and 89.7% in people aged 70 or over. Please note that the model is unable to assess the effect of face coverings of children due to lack of data but is able to assess the effect of adults wearing face coverings on transmission in children.

For completeness, a summary of the model parameters is provided in online supplemental table 1. This table lists the acronym of each parameter, along with the prior and posterior densities. If a parameter is not shared by all age groups, the densities for the first age group are shown. These (lognormal) densities are summarised in terms of their expected value and 90% credible intervals. A lognormal distribution precludes negative values for a parameter. Practically, this means one estimates the logarithm of a parameter, under the assumption the log transformed parameter has a normal or Gaussian distribution.

A brief description of each parameter is also provided. The prior values were chosen using the best empirical evidence at the time the model was constructed, and subsequently revised using Bayesian model comparison, as described in Friston *et al*.^3^ The prior and posterior expectations correspond to the mean value. Because the probability densities had a lognormal form, the mean is also the mode; that is, the most likely value.

Almost universally, model parameters correspond to the probability of moving from one state to another, per day. These rates can either be expressed in terms of probabilities or their inverse; namely, the average number of days spent in a particular state, before moving to another state. Probabilities or proportions that exceed one are transformed to lie in the range [0,1], using the error function. The initial (prior) values of the parameters mediating the effects of season (number 16 in online supplemental table 1) and face covering (number 61 in online supplemental table 1) were set to 0.04 (4%) with a mildly informative prior variance.

#### Data sources

The data series available in November 2023 included UK government sourced data from the Office of National Statistics (ONS) and the UK Health Security Agency (UKHSA) and mobility data from Google.^12 13^ An additional series of data from May 2020 to April 2023 providing self-reported use of face coverings by adults (over 15 years of age) in public from ONS^14^ was incorporated into the model to create M(*t*) by interpolating the biweekly data into a daily timeseries. The question on face covering use was first added to the ONS Opinions and Lifestyle Survey on 29 May 2020 and repeated—usually fortnightly—over the study period with the question:

In the past 7 days, have you used a face covering when outside your home to slow the spread of the coronavirus (COVID-19)?

Unfortunately, no data were collected and therefore available for use in the analysis on face coverings among children. In addition, there were no data available on the type of face covering worn or when or where or the consistency of face covering. And—while the survey was of a high standard with ONS providing detailed quality and methodology information—the question on the use of face covering was not externally validated.^15^ An external CORSAIR study in the UK did identify the type of face covering used in May 2020, reporting that 30% of respondents—who had worn a face mask outside, during the coronavirus pandemic—wore a medical mask that they had bought. Furthermore, 26% had been wearing a homemade cloth mask, while 23% wore a cloth face mask that they purchased, 17% a scarf and 12% other coverings.

## Results

**Figure 2:**
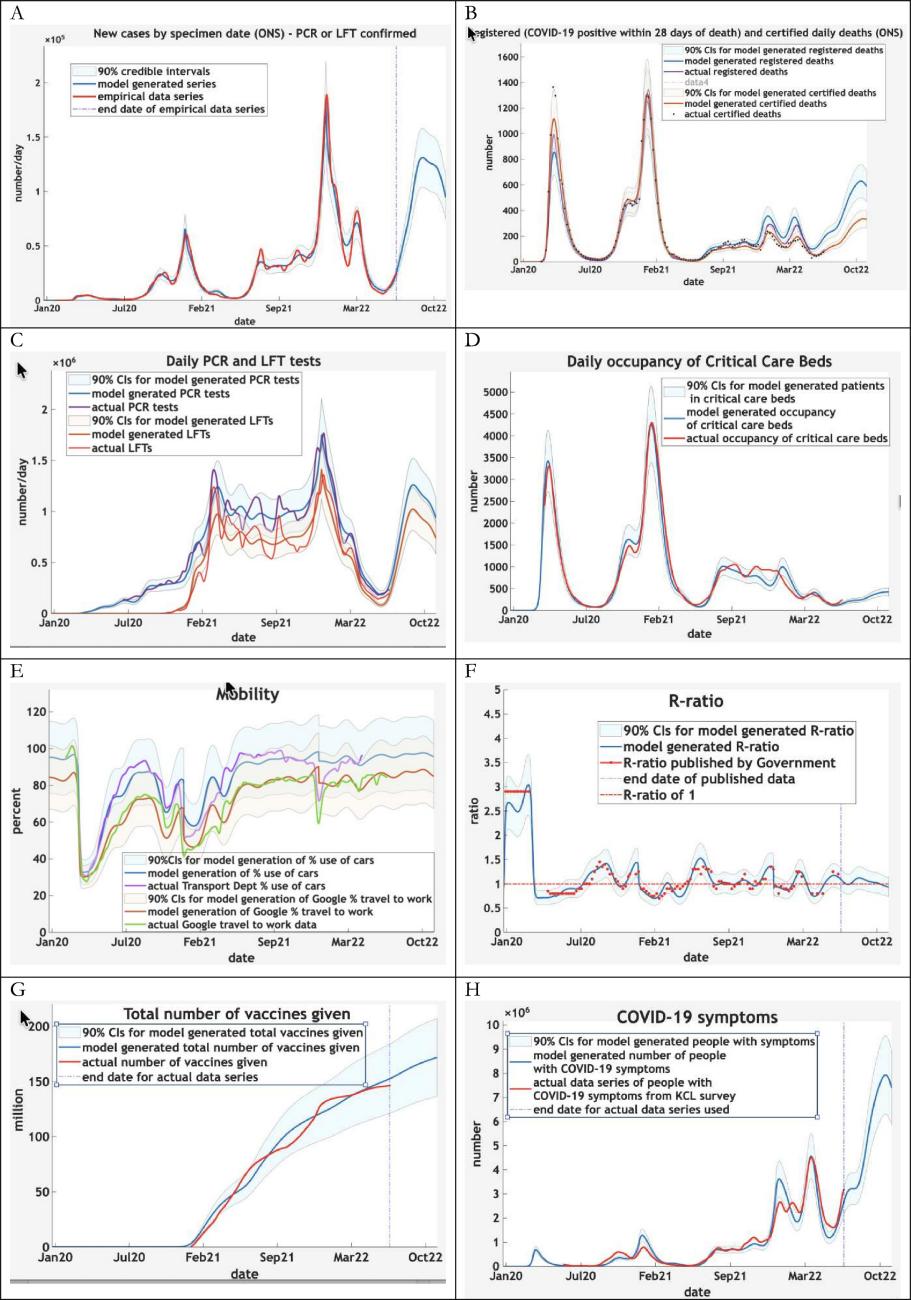
Comparison of COVID-19-related data with predictions.These graphs show the predicted outcomes from January 2020 to October 2022 UK of some selected outcomes. The predictions are based upon latent population states inferred after model fitting (i.e., the posterior estimates shown in the subsequent figure). The key thing to take from these results is the ability to provide a fairly accurate account of multiple aspects of the epidemic, in terms of the underlying causes (i.e., latent states) generating empirical data. The coloured lines correspond to posterior expectations, while the shaded areas correspond to 90% credible intervals. The red lines and dots are the empirical time-series used to estimate the model parameters and ensuing time-dependent states. KCL, Kings College London; LFT, lateral flow test; ONS, Office of National Statistics.

The model was fit to the above data over a 30-month period from the onset of the pandemic (1 January 2020). This period covered the first two waves and subsequent fluctuations, noting that face covering increased following the first wave due to the availability of face coverings and governmental advice, and later mandatory legal requirements. Figure 2 and online supplemental figure 1 illustrate the accuracy of the model in terms of actual data (selected from over 40 different kinds of data). The model can be seen to explain a wide variety of data fairly accurately. Online supplemental figure 2 shows the corresponding latent states (see table 1) generating these data, partitioned into their respective factors. For example, there are eight (five unvaccinated plus three vaccinated) possible states of ‘infection’ with the probability of being in any one state changing with time as the epidemic progresses.

**Figure 3:**
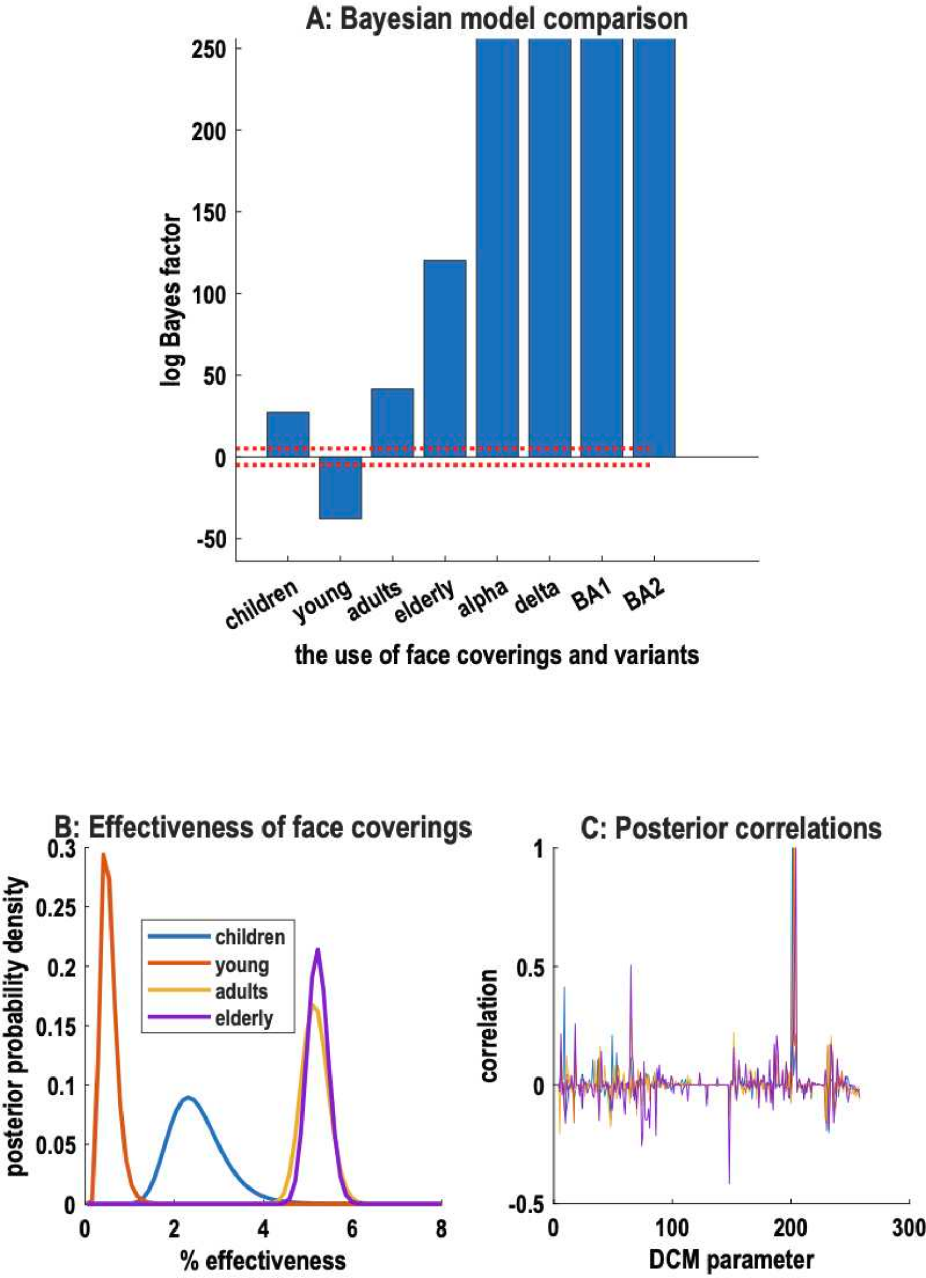
The evidence for face covering. (A) reports the differences in log evidence when comparing a model with and without a particular effect. The first four columns correspond to the effectiveness of mask-wearing for the four age groups used in the age-stratified DCM. The last columns pertain to increases in transmissibility or transmission risk incurred by the emergence of new variants. This graph has been truncated at 256 natural units. The red dotted lines correspond to plus and minus 5 natural units. This is normally taken as a threshold to declare that there is very strong evidence for one model over another.^25^ We see here that there is very strong evidence for an effect of mask-wearing on children, and older adults but evidence for a trivial effect on younger adults. (B) reports the posterior probability densities over the effectiveness for each of the four age groups. (C) reports the correlations found between all the model parameters and each age-specific effect of the face covering parameter. The peaks with correlations of 1 are the auto-correlations of the mask parameters with themselves. All other parameters have correlations below 0.5 reflecting mild conditional dependencies. In short, the effect of mask-wearing can be disentangled from other effects. DCM, dynamic causal modelling.

Figure 3 reports the evidence of an effect of face covering in the adult population on each of four age groups. This evidence was evaluated using Bayesian model reduction (see table 1).^16^ The first four columns in figure 3A present the difference in log evidence of models with and without face coverings for the four age groups. This difference corresponds to a log Bayes factor. If the log Bayes factor is greater than 3, then one can assert there is strong evidence for the model with an effect.^17^ This is because exp(3)≈20, implying that the (full) model with an effect is 20 times more likely than the (reduced) model without an effect. Here, the reduced models assumed that the effect of face covering was trivial; by reducing the prior expectation by two orders of magnitude (ie, 100-fold). For comparison, we also compared models with and without increases in transmissibility due to the emergence of new variants: see columns 5–8 of figure 3A.

Interestingly, there is very strong evidence for an effect of face covering on adults and the elderly but weaker evidence for an effect on children and contrary evidence for younger adults. In fact, the reduced model—with no effect of face covering on younger adults—had more evidence than a model with an effect. In other words, a model with no effect of face covering on younger adults provides a better explanation for the data. This can be seen in figure 3A as a negative difference of greater than 5 (lower dotted line), indicating very strong evidence for the absence of a face covering effect in this group. Conversely, one can be almost certain there was an effect of face covering in the remaining three age groups. This is reflected in figure 3B in terms of the posterior densities over the effectiveness of face covering in the four groups. Posterior densities represent the updated probability density of an unknown variable (eg, parameter) after observing data. This probability density reflects the ‘belief’ about the parameter having seen the data, in contrast to the prior distribution, which represents the initial belief before observing the data. One can see that the effectiveness was about 5% for adults and elderly age groups, while there was a small effectiveness for children. Specifically, the means (and 90% credible intervals) for children (less than 15 years), young adults (15–34 years), adults (35–69 years) and the elderly (70 years or more) were: 2.3% (90% CI 1.5% to 3.4%), 0.4% (90% CI 0.2% to 0.8%), 5.1% (90% CI 4.6% to 5.6%) and 5.2% (90% CI: 4.8% to 5.6%), respectively.

Figure 3C reports the posterior correlations between the effect of face covering and remaining model parameters to show that these correlations were minimal, that is, that the effect of face covering could be disentangled from other effects, including the sociobehavioural responses to prevalence and other factors. Finally, one might ask what would have happened in the absence of any face covering?

**Figure 4:**
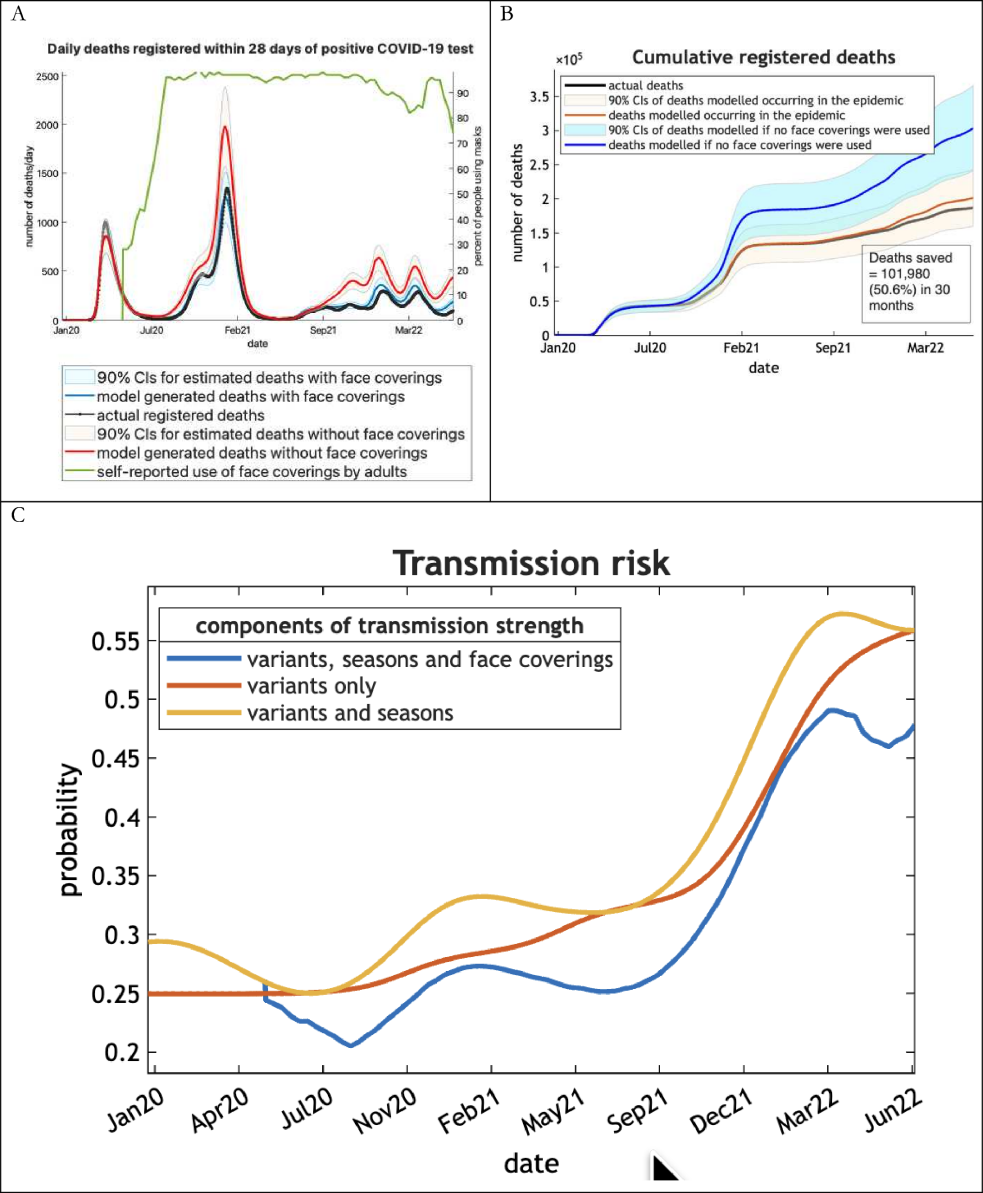
Registered deaths with and without face coverings and fluctuations in transmission strength from January 2020 to June 2022, UK. (A, B) show the evolution of registered deaths and (B) shows the evolution of registered deaths as a function of time during the 30-month analysis period. The blue areas and line report the 90% Bayesian credible intervals and estimates with the inclusion of face covering, while the red areas and line show the increase in mortality if face coverings were suppressed. The black line corresponds to actual data. Note that the Bayesian credible intervals do not overlap, suggesting the effect size (in terms of predictive posteriors) was substantial, even when accounting for uncertainty. Interestingly, the effect of face coverings is not to just attenuate the amplitude of peak mortalities but to defer or delay the onset of a wave, as would be expected given the underlying dynamics. (C) reports the contributions to changing transmission strength or risk from January 2020 to June 2022 in the UK. The red line is the underlying transmission risk due to different variants, while the yellow line adds seasonality and the third, blue line includes the face covering effect. This illustrates the relative contribution of seasonal and behavioural fluctuations superimposed upon a progressive rise in transmissibility due to the emergence of successive variants.

One can address this question by re-running the model in the absence of any face covering while keeping all the other parameters (ie, rates, efficacies and sensitivities) the same. Figure 4A shows the results of this analysis for mortality (ie, registered deaths due to COVID-19) as a function of time. It suggests a marked impact of face covering: although the effectiveness of face covering only reduced transmission risk by a few percent, the number of lives saved could have been many tens of percent. In this analysis, over the 30-month period, about 102 000 (90% credible interval range between 40 000 and 164 000) people could have died without face covering (figure 4B). This corresponds to a 50% increase in mortality burden.

These results should not be overinterpreted because one cannot know how people would have responded in the absence of face covering; however, this quantitative analysis speaks to a clear effectiveness of face covering in reducing community transmission. Furthermore, these retrospective modelling results endorse the observations of an early modelling study of face covering in the US states of New York and Washington from which the authors conclude ‘mask use decreases the effective transmission rate in nearly linear proportion to the product of mask effectiveness and coverage rate, while the impact on epidemiologic outcomes (death, hospitalisations) is highly non-linear, indicating face coverings could synergise with other non-pharmaceutical measures’.^18^

The effect of face coverings on community transmission is shown in figure 4C in terms of the relative contribution of new variants, seasonality and face coverings. Here, the effect of winter surges is superimposed on the increase in transmission strength due to the advent of new variants. Transmission strength is reduced by about 5% when the effect of face coverings is included.

## Discussion

Our modelling suggests that—within the 30-month period analysed, when face coverings were used by the majority of adults and older school children—face coverings had a significant effect on transmission (reduced by 5%) and a more dramatic effect in reducing mortality, perhaps by as much as 50%. The effect is most evident in the two older age groups studied (ie, people aged 35 or more).

Although the age-dependent transmission risk effects are interesting, the modelling offers no mechanistic explanation for these effects. One could speculate that the effectiveness of face coverings depends on the context in which they are worn. For example, young adults may tend to work more in outdoor environments—which might attenuate the effect of face coverings on aerosol transmission—relative to working in offices and residential homes. To test this (and many other hypotheses), one would probably require stratified data or randomised controlled trials (RCTs). Perhaps the more interesting question here is not why the effectiveness of face coverings is age-dependent, rather what causes different groups to wear face coverings. This will be the focus of a subsequent DCM study, in which face covering is treated as a consequence, as opposed to a cause of underlying behavioural, clinical and epidemiological states.

The model provides an accurate explanation for multiple data provided by governmental and other institutions. Furthermore, the DCM used in this modelling has an established predictive validity. This suggests the parameter estimates relating to the use of a face covering are reasonably accurate and reliable. In the review and meta-analysis of RCTs of face covering in a community setting which motivated the current study, the only trials to show a significant effect of face covering used clinical measures of influenza and COVID-19 like symptoms.^1^ These RCTs suggested a relative risk or risk ratio (RR) of 0.89 (95% CI 0.87 to 0.91). Trials using PCR-confirmed cases of influenza and SARS-CoV-2 suggest an average effectiveness of about 14%, with a RR of 0.86 (95% CI 0.5 to 1.46). However, the CI here contains a relative risk of unity, meaning that one cannot reject the hypothesis that face coverings have no effect. In contrast, the equivalent relative transmission risk, estimated by DCM, suggests that (for adults) we can be almost certain there is a non-trivial effect of face coverings: 5.1% (90% CI 4.6% to 5.6%). This translates into a credible RR interval of 0.944–0.954, comfortably within the RCT CIs.

The differences between the confidence placed in estimates of effectiveness may reflect the differences in statistical power of RCTs vs community or population modelling. The current modelling could be viewed as leveraging a natural (ie, uncontrolled) experiment that fortuitously generated data for the first wave in the absence of face covering and a second wave with face covering. Furthermore, the onset of face covering predated the emergence of the first (alpha) variant. This sequence of events allowed the model to disentangle the effects of face covering on transmission—using a vast amount of data—to provide a very precise estimate of effectiveness in terms of relative risk. One might argue that not only was the data generated by the COVID-19 epidemic unprecedented, but it may never be repeated. In other words, the opportunity for the kind of modelling described above may be unique.

Having said this, RCTs have certain advantages over population modelling. For example, the DCM estimates of effectiveness lumped together different kinds of face coverings—and are averaged over everyone in the population (within the age groups considered). In other words, although one can be confident that face covering—in at least one age cohort—had a non-trivial effect on community transmission, it is more than likely that with particular face coverings and in particular (eg, medical) settings their effectiveness would be higher. Similarly, in other settings, they may have no effect (eg, working outdoors). The quality of mask design and face coverage in the UK, over the period considered, would have been mixed. We were using self-reported use in a national survey—of many features of living associated with the epidemic—to assess the use of face coverings. While the face covering survey used was of high quality and methodologically sound, external validity was not assessed. We do not know what aspects of face covering produced the estimated 5% reduction in transmission. All we can infer is that face covering, on average, reduced transmission risk with a substantial reduction in mortality. In respect of previous studies, while RCT meta-analyses could be considered reasonable in theory, the DCM results can be considered useful in practice.

Over the first 4 years of the pandemic, more than 235 000 deaths have been attributed to COVID-19 in the UK. At the initial press conference on 12 March 2020, the journalist Beth Rigby asked Professor Whitty “… can you level with us and say how many people will actually die?” Whitty said, “In terms of being level, our top planning assumption is up to 80% of population will be infected, and the mortality rate is in our view 1% or less overall, higher in elderly groups.” These assumptions suggested that in the UK, 500 000 or more people could die in the absence of interventions. The lower death rate of 235 000 may, therefore, be partly explained by face covering on a large scale, and 102 000 deaths prevented by face coverings may not be an overestimate. Although avoiding an excess mortality of 50% may sound large, it is in line with early prospective scenario modelling of the use of face coverings. For example, ‘Even very weak masks (20% effective) can still be useful if the underlying transmission rate is relatively low or decreasing: in Washington, where baseline transmission is much less intense, 80% adoption of such masks could reduce mortality by 24%–65% (and peak deaths 15%–69%)’.^18^

In summary, based on our results, face coverings, even of the type and mixed quality used in the UK, seem to have had a large cumulative effect on COVID-19-related deaths, and perhaps more than expected. This was despite a small reduction in transmission risk of about 5% over this period. Our findings support the ‘prevention paradox’ first proposed by Professor Geoffrey Rose, which explained how simple interventions in a low-risk population with potentially little individual benefit could have a much larger impact on population benefit than interventions focused only on higher risk individuals.^19^

Other interventions to suppress the pandemic early on, through rapid case finding, expansion of testing in hotspot areas, mobilisation of community health workers to find cases and support isolation, and generous financial support to ensure high compliance with 14 days of isolation, produced dramatic reductions in mortality in East Asia. The UK and many other Western countries, however, did not do this successfully.^20^ We have studied the potential effect of an improved case finding programme in the UK using our model.^8^ It could have had a dramatic effect on outcomes. It would be possible to run similar scenarios based on East Asian type interventions and study the effect of face coverings in these settings. It is also straightforward to assess the effect of face covering on other COVID-19 outcomes such as hospitalisation, severe cardiovascular morbidity and other post-COVID-19 syndromes. The DCM is available—as open-source software—for infectious disease research groups to explore this further.

From a modelling or technical perspective, DCM offers an efficient and reproducible alternative to conventional epidemiological and agent-based modelling. DCM is a mainstream procedure in medical (eg, brain imaging) timeseries analysis^21^ that was first applied to infectious disease modelling during the COVID pandemic.^6^ It offers several advantages over conventional approaches. For example, instead of simulating the stochastic behaviour of multiple agents numerically, it models the probability distribution over a population of agents analytically. In other words, it considers the solution to population density dynamics in the limit of a large number of stochastic agents. The requisite probability density dynamics—that describe fluctuations in the sociobehavioural and clinical states of a population—rest on unknown model parameters that are estimated from data.

There are two ways of estimating these parameters (and quantifying uncertainty about these estimates). One can use sampling procedures such as MCMC, Gibbs sampling or Metropolis Hastings to approximate the posterior density numerically.^22^ Alternatively, one can assume a functional form for the posterior density and use variational procedures, such as Variational Laplace.^23 24^ DCM uses Variational Laplace to estimate model parameters under the assumption they have a normal—or lognormal—form. This has some fundamental advantages over sampling schemes. For example, the posterior estimates are exactly reproducible, in the sense the same results are always obtained given the same data. Furthermore, there are no tuning parameters or hyper-parameters that need to be specified: every parameter and hyperparameter is uniquely specified as that which extremises variational free energy (a.k.a. an evidence lower bound in machine learning).^24^ Similarly, the convergence of variational schemes is assessed in a straightforward way (ie, when the variational free energy stops changing).

In addition to the computational and statistical efficiency of variational procedures, there is an important benefit over sampling procedures; namely, the variational free energy provides a bound on model evidence (a.k.a., marginal likelihood). This affords the opportunity to compare different models or optimise model structure as more data becomes available. This kind of model optimisation rests on something called Bayesian model comparison.^25^ Please see Friston *et al*^2^ for a discussion of Bayesian model comparison and the DCM for COVID-19. For readers interested in the foundational technical details,^23^ provides a nice introduction to variational Laplace. For people interested in the technical details of the DCM used in this paper, there is a comprehensive series of reports detailing its construction,^4 6 26 27^ evolution^2 5 28^ and validation.^8^,^10^ The accompanying data and software are available as open source for people wishing to reproduce or extend the analyses reported in the current paper.

There are limitations to the DCM approach: please see references^2^,^5^ for a fuller discussion of the caveats and limitations of DCM. For example, removing the effect of face covering from the model does not consider consequent changes in other parameters. We do not know how people would have responded in the absence of face covering. We have found clear age differences that have not been explained mechanistically. In particular, we do not know the effect of children wearing face coverings on transmission. This requires further analysis. However, overall, this quantitative analysis speaks to a clear effectiveness of face covering in reducing community transmission.

## Supplementary material

10.1136/bmjph-2025-003489 online supplemental file 1

10.1136/bmjph-2025-003489 online supplemental file 2

10.1136/bmjph-2025-003489 online supplemental file 3

10.1136/bmjph-2025-003489 online supplemental file 4

## Data Availability

Data are available on reasonable request.
