# Supplementary material for: Estimating the value of face coverings during the COVID-19 epidemic: a dynamic causal modelling study
Source: BMJ Public Health. 2025 Dec 25;3(2):e003489. doi: 10.1136/bmjph-2025-003489 (PMC12742113; doi:10.1136/bmjph-2025-003489)
Supplement: online supplemental file 1 [file bmjph-3-2-s001.docx]

## **Supplement on data series and software to help reproduce the findings**

There are two sources of the DCM Covid-19 model. The chief source is annotated (MATLAB) code available as part of the open-source academic software SPM available at <https://www.fil.ion.ucl.ac.uk/spm/covid-19/>. An additional source is available at <https://www.dropbox.com/scl/fo/4d6sagxchp5mqybrwj2l8/AERr4owH1vnGilrhUVtKQ7g?rlkey=e82lqft21nyzl2s32pag11gc3&st=czsipr3h&dl=0> for this research paper only. The folder contains the following code and subroutines:-

|  | DEM_COVID_MASKS.m |
| --- | --- |
|  | DEM_COVID_UK4.m |
|  | spm_SARS_gen.m |
|  | spm_SARS_plot.m |
|  | spm_SARS_priors.m |
|  | spm_COVID_T.m |
|  | DEM_masks_Cam.m |
|  | spm_plot_ci_cam.m |
| Karl_DEM | spm.m |
| Karl_DEM | spm_COVID_Y.m |
| Karl_DEM | spm_COVID_table.m |
| Karl_DEM | spm_SARS_ci.m |
| Karl_DEM | spm_axis.m |
| Karl_DEM | spm_cat.m |
| Karl_DEM | spm_check_version.m |
| Karl_DEM | spm_cross.m |
| Karl_DEM | spm_defaults.m |
| Karl_DEM | spm_diag.m |
| Karl_DEM | spm_fieldindices.m |
| Karl_DEM | spm_figure.m |
| Karl_DEM | spm_get_defaults.m |
| Karl_DEM | spm_invNcdf.m |
| Karl_DEM | spm_kron.m |
| Karl_DEM | spm_length.m |
| Karl_DEM | spm_marginal.m |
| Karl_DEM | spm_permute_kron.m |
| Karl_DEM | spm_phi.m |
| Karl_DEM | spm_platform.m |
| Karl_DEM | spm_plot_ci.m |
| Karl_DEM | spm_sum.m |
| Karl_DEM | spm_unvec.m |
| Karl_DEM | spm_vec.m |
| Karl_DEM | spm_vecfun.m |
| Karl_DEM | spm_zeros.m |

The following data files are required which can be found in the following sources or in the data_series.zip file.

: <https://coronavirus.data.gov.uk>; <https://www.ons.gov.uk/peoplepopulationandcommunity/healthandsocialcare/conditionsanddiseases/datasets/coronaviruscovid19infectionsurveydata>; [https://covid.joinzoe.com/data#levels-over-time](https://covid.joinzoe.com/data%2523levels-over-time); [https://www.gov.uk/guidance/the-r-number-in-the-uk#contents](https://www.gov.uk/guidance/the-r-number-in-the-uk%2523contents); <https://www.gov.uk/government/statistics/transport-use-during-the-coronavirus-covid-19-pandemic>; <https://www.google.com/covid19/mobility/>.

| admissions.csv |
| --- |
| agecases.csv |
| agedeaths.csv |
| agevaccine.csv |
| cases.csv |
| certified.csv |
| critical.csv |
| cumAdmiss.csv |
| deaths.csv |
| gdp.csv |
| lateralft.csv |
| mobility.csv |
| occupancy.csv |
| place.csv |
| positivity.csv |
| ratio.csv |
| serology.csv |
| survey.csv |
| surveyage.csv |
| symptoms.csv |
| tests.csv |
| transport.csv |
| vaccines.csv |

One way to run the full model is to launch DEM_COVID_UK4.m in Matlab.

DEM_COVID_UK4.m Demonstration of COVID-19 modelling using variational Laplace (4 groups)

%_________________________________________________________________________

This routine illustrates the dynamic causal modelling of the epidemic in the United Kingdom using four age groups that are coupled via (prevalence -dependent) contact rates. It is the routine used to prepare the graphics and report for the DCM COVID dashboard.

- It sets up the data, most easily done with historical data importing from the datafiles listed above
- It creates a data structure
- It cleans and sorts the data
- It gets the prior parameters from spm_SARS_priors
- It prepares the model specification which uses two key programmes – spm_SARS_gen.m which generates predictions and hidden states of the COVID model and which uses spm_COVID_T, which creates transition probability tensors as a function of the model parameters and the joint density over four factors, each with several levels
- It undertakes model inversion with Variational Laplace (Gauss Newton). This takes about 12 hours on a fast Mac. To bypass this stage you can use the model already made and saved in DCM_UK_tmp.mat
- It saves the model
- It unpacks the model with the newly chosen posterior parameters
- It undertakes posterior predictions and creates the routine graphs some of which are shown in the manuscript.

To provide the special figures created for the paper run DEM_masks_Cam.m which

- Applies Bayesian model reduction to the DCM asking whether mask wearing can be treated as fixed parameters by reducing its prior variance to 0
- Displays the results of Bayesian model comparison
- Plots epidemiological trajectories of registered deaths with or without masks
- Plots cumulative deaths with or without masks
- Plots transmission strengths.

Advice can be obtained by contacting Cam Bowie at [cam.bowie1@gmail.com](mailto:cam.bowie1@gmail.com).
