# Supplementary material for: Estimating the value of face coverings during the COVID-19 epidemic: a dynamic causal modelling study
Source: BMJ Public Health. 2025 Dec 25;3(2):e003489. doi: 10.1136/bmjph-2025-003489 (PMC12742113; doi:10.1136/bmjph-2025-003489)
Supplement: online supplemental file 2 [file bmjph-3-2-s002.docx]

**Supplement Figure 1 – Results of the modified DMC model which includes mask use**

**
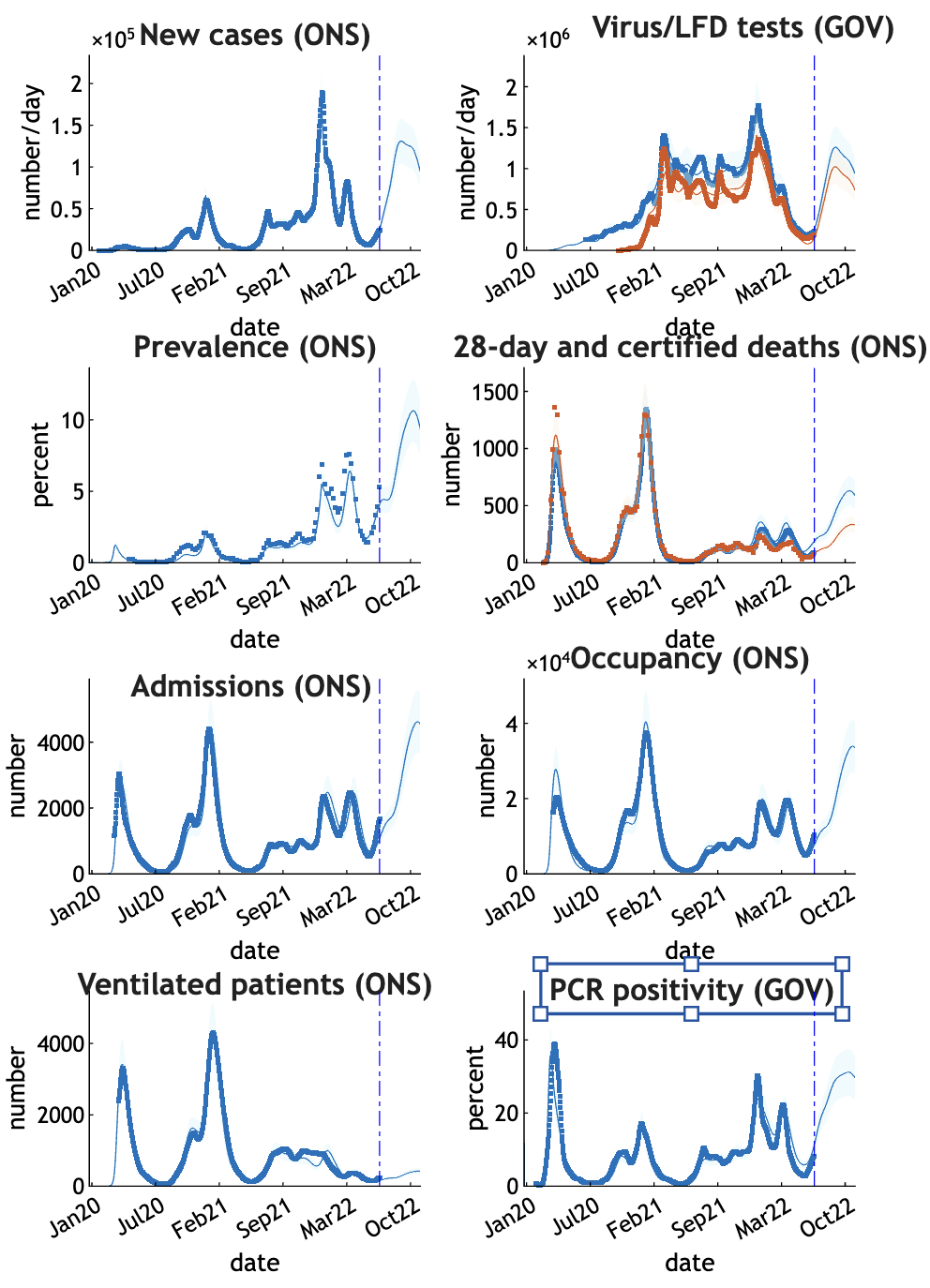
**

**
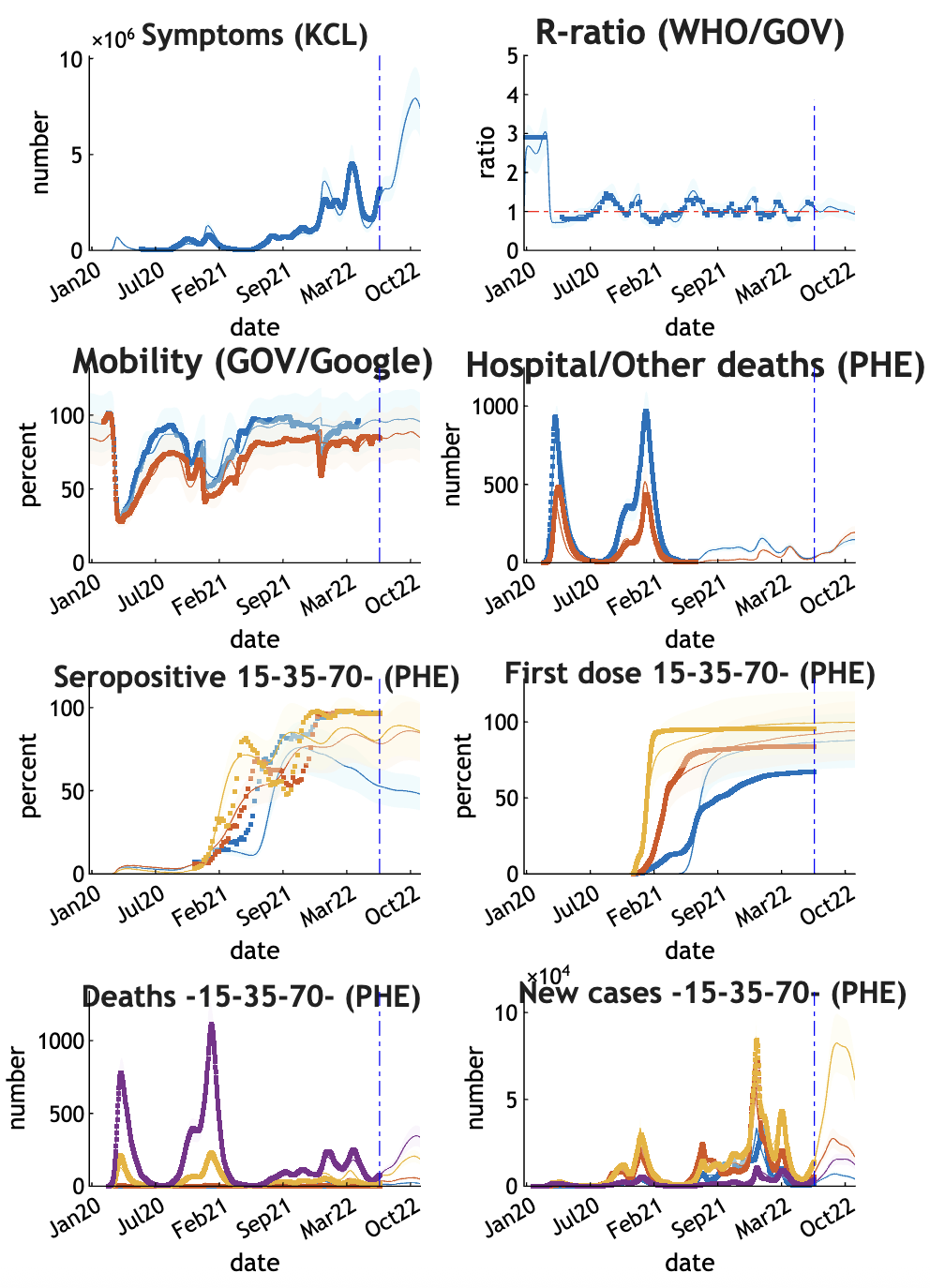
**

**Supplement Figure 1: A comparison of COVID–19 related data with predictions**. These graphs show the predicted outcomes from January 2020 to October 2022 UK. The predictions are based upon latent population states inferred after model fitting (i.e., the posterior estimates shown in the subsequent figure). The key thing to take from these results is the ability to provide a fairly accurate account of multiple aspects of the epidemic, in terms of the underlying causes (i.e., latent states) generating empirical data. The coloured lines correspond to posterior expectations, while the shaded areas correspond to 90% credible intervals. Thick lines or dots are published data; thin lines and shades are Dynamic Causal Model estimates with 90% Bayesian credible intervals. GOV relates to NHS, Department of Health and Department of Transport published data, KCL to their Covid Health Study, PHE to Public Health England, and ONS to Office of National Statistics. Mobility GOV in blue; Google in orange. Hospital deaths in blue; other deaths in orange. Seropositive and vaccine coverage by 3 age group - blue 15–34 years, orange 35–69 years, yellow 70+ years. Deaths and new cases by 4 age groups – blue 0-14 years, orange 15-34 years, yellow 35-69 years, purple 70+ years.
