# Supplementary material for: Estimating the value of face coverings during the COVID-19 epidemic: a dynamic causal modelling study
Source: BMJ Public Health. 2025 Dec 25;3(2):e003489. doi: 10.1136/bmjph-2025-003489 (PMC12742113; doi:10.1136/bmjph-2025-003489)
Supplement: online supplemental file 3 [file bmjph-3-2-s003.docx]

*
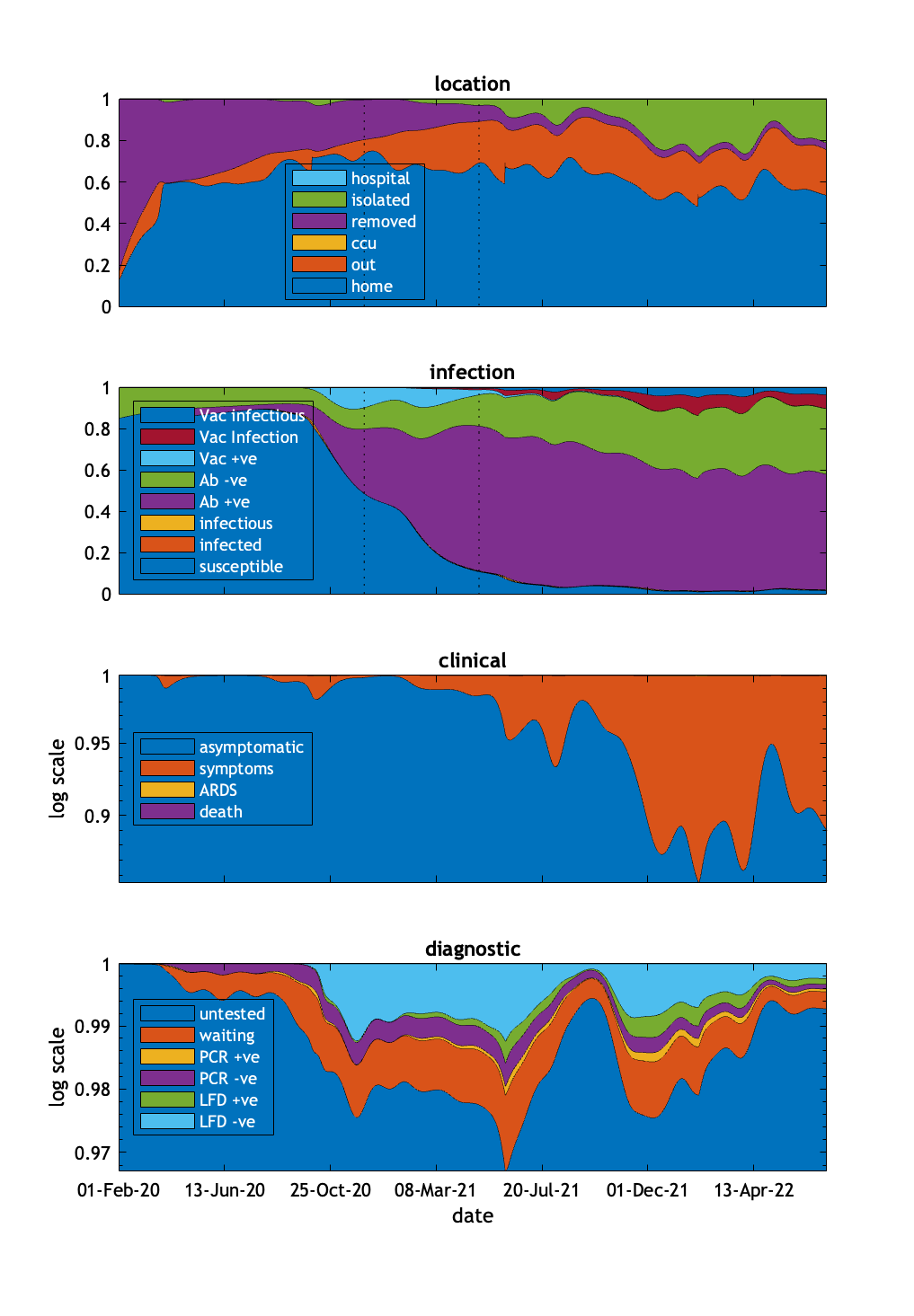
*

**Supplement Figure 2** – **Latent states.** These graphs report the proportion of the population in the various states of the four factors in Figure 1 over the period January 2020 to October 2022 UK. The proportion of the population in each state generates the predicted outcomes shown in the previous figure. The y-axis of each figure is the probability of an individual being in a particular state, when sampled at random from the population (which sum to one). This is the same as the proportion of the population in a particular state, at any given time. Note the y-axis of the third and fourth figures are truncated log scales. Please see for further explanation of the latent states and their interpretation (3).
