# Supplementary material for: Estimating the value of face coverings during the COVID-19 epidemic: a dynamic causal modelling study
Source: BMJ Public Health. 2025 Dec 25;3(2):e003489. doi: 10.1136/bmjph-2025-003489 (PMC12742113; doi:10.1136/bmjph-2025-003489)
Supplement: online supplemental file 4 [file bmjph-3-2-s004.pdf]

Supplement Table 1

Parameters used in the model			Initial (prior) parameters			(Posterior) parameters chosen by the model			Comments
number	name	description	mean	lower bound	upper bound	mean	90% credible intervals		
1	n	population size (Million)	12.0065	12.0065	12.0065	12.0065	12.0065	12.0065	size of a large region
2	n	initial cases	8.39E-05	1.62E-05	4.34E-04	3.67E-09	2.67E-09	5.06E-09	a few individuals
3	r	pre-existing immunity	0.1	0.09	0.11	0.14	0.13	0.15	pre-existing immunity due to previous SARS-CoV infection
4	o	initially exposed	0.1	0.08	0.12	0.06	0.05	0.07	proportion of people initially exposed
5	m	relative eflux	0.1	0.08	0.12	0.06	0.05	0.07	proportion leaving the population
6	out	P(leaving home)	0.4	0.37	0.43	0.40	0.38	0.43	proportion leaving home each day
7	sde	time constant of lockdown	4	3.20	5.00	4.44	3.55	5.54	individual sensitivity to high prevalence by staying at home
8	qua	time constant of unlocking	128	118	139	274	264	283	individual sensitivity to low prevalence to leaving home
9	exp	viral spreading (days)	0.02	0.016	0.025	0.028	0.024	0.033	exposure time required to cause infection
10	hos	admission rate (hospital)	2	1.60	2.50	2.28	1.82	2.84	admission rate to hospital
11	ccu	admission rate (critical)	0.2	0.16	0.25	0.33	0.27	0.40	admission rate to an intensive care bed
12	s	infectivity changes	1	0.92	1.09	1.21	1.12	1.31	dummy for time dependent parameters such as infectiousness of new variants
13	nin	contacts: home	1	0.92	1.09	0.87	0.80	0.94	the number of contacts of people at home for the day
14	nou	contacts: work	16	14.74	17.37	14.85	13.95	15.81	the number of contacts of people who leave home each day
15	trn	transmission strength	0.2	0.18	0.22	0.20	0.19	0.21	transmission strength of the virus on any day
16	trm	seasonal transmission	0.04	0.039	0.041	0.044	0.043	0.045	the strength of the seasonal effect on transmission
17	tin	infected period (days)	3	2.91	3.09	2.61	2.54	2.69	pre-infectious period or "latent period"
18	tcn	infectious period (days)	4	3.88	4.12	4.09	3.99	4.20	infectious period including pre-symptomatic and symptomatic periods
19	tim	loss of natural immunity (days)	128	118	139	143	133	153	loss of b-cell immunity in days
20	res	resistance	0.2	0.18	0.22	0.14	0.13	0.15	inherent immunity to infection
21	tic	asymptomatic period (days)	4	3.69	4.34	3.25	3.07	3.43	period between onset of infection and symptoms - incubation period
22	tsy	symptomatic period (days)	5	4.61	5.43	5.77	5.56	5.99	period between start and end of symptoms
23	trd	critical period (days)	16	12.8	20.0	9.52	9.24	9.81	duration of ARDS (Acute Respiratory Distress Syundrome)
24	sev	P(ARDS|symptoms): initial	0.002	0.0011	0.0037	0.0068	0.0058	0.0080	proportion of symptomatic patients who develop ARDS at the start of the epidemic
25	lat	P(ARDS|symptoms): change	1	0.55	1.83	2.33	2.23	2.43	proportion of symptomatic patients who develop ARDS as the epidemic progresses
26	fat	P(fatality|ARDS): initial	1.00E-06	5.46E-07	1.83E-06	1.00E-06	5.46E-07	1.83E-06	proportion of ARDS patients who die at the start of the epidemic
27	sur	P(fatality|ARDS): change	1	0.55	1.83	2.86	2.70	3.02	proportion of ARDS patients who die as the epidemic progresses
28	ttt	FTTI efficacy	0.036	0.029	0.045	0.036	0.029	0.044	the effectiveness of the "find, test, trace, isolate" process
29	tes	testing: bias (PCR)	16	8.74	29.30	2.97	1.62	5.44	PCR testing rate of infected cases
30	tts	testing: bias (LFD)	1	0.55	1.83	2.29	1.25	4.19	LFT testing rate of infected cases
31	del	test delay (days)	3	2.76	3.26	3.08	2.84	3.34	delay between test taken and result

Parameters used in the model			Initial (prior) parameters			(Posterior) parameters chosen by the model			Comments
number	name	description	mean	lower bound	upper bound	mean	90% credible intervals		
32	vac	vaccine seroconversion (days)	32	29.5	34.7	36.76	34.20	39.50	period from date of vaccination to protection from infection
33	fnr	false-negative rate	0.08	0.074	0.087	0.051	0.047	0.055	sensitivity of PCR test
34	fpr	false-positive rate	0.0002	0.00018	0.00022	0.00021	0.00019	0.00023	specificity of PCR test
35	lim	testing: capacity	0.0005	0.00027	0.00092	0.00134	0.00085	0.00212	testing capacity
36	rat	testing: constant	8	7.37	8.68	8.41	7.75	9.13	growth of testing capacity
37	ons	testing: onset	100	19	518	326	306	347	start of testing
38	lag	reporting lag	1	1	1	1	1	1	lag in reporting results
39	inn	seasonal phase	1	0.55	1.83	6.73	4.23	10.71	seasonal variation in transmission risk
40	mem	vaccination rollout (days)	128	124	132	133	129	136	speed of vaccination roll out
41	rol	vaccination rollout (1st)	0.0001	6.63E-05	0.000151	0.000103	6.8E-05	0.00015	start of first roll out phase of vaccinations
42	fol	vaccination rollout (2nd)	0.0001	6.63E-05	0.000151	0.000109	7.2E-05	0.00016	start of second roll out phase of vaccinations
43	vef	vaccine efficacy: sterilising	0.4	0.37	0.43	0.73	0.69	0.78	vaccine efficacy producing inability of the virus to infect susceptible people
44	lnk	vaccine efficacy: pathogenicity	0.24	0.24	0.24	0.24	0.24	0.24	vaccine efficacy preventing clinical disease
45	ves	vaccine efficacy: transmission	0.1	0.092	0.109	0.385	0.365	0.407	vaccine efficacy preventing transmission of the virus from an infected person
46	lnf	vaccine efficacy: fatality	0.05	0.046	0.054	0.122	0.114	0.131	vaccine efficacy preventing death
47	con	LFD confirmation	0.2	0.11	0.37	0.13	0.07	0.24	the proportion of LFT positive tests confirmed by a PCR test
48	iso	self-isolation (days)	8.0	7.8	8.2	8.3	8.1	8.6	the period in which an individual isolates
49	tnn	loss of T-cell immunity	256	248	264	254	247	262	loss of T-cell (antibody negative) immunity in days
50	lnr	LFD specificity	0.46	0.42	0.50	0.67	0.62	0.73	specificity of a LFD test
51	lpr	LFD sensitivity	0.0002	0.00018	0.00022	0.00020	0.00018	0.00021	sensitivity of a LFD test
52	rel	PCR testing of fatalities	1	0.80	1.25	1.44	1.23	1.68	the proportion of COVID-19 related deaths which are untested
53	pro	contact rate decay (days)	1	0.80	1.25	23.91	22.53	25.38	the change in contact rates due to a changing sensitivity to prevalence
54	oth	survival risk in care homes	0.1	0.08	0.12	0.08	0.07	0.10	a change in survival risk of ARDS cases such as for those in care homes
55	iad	changes in transfer to CCU	1	0.55	1.83	2.03	1.92	2.14	a change in the admission rate to intensive care beds
56	tra	transmissibility parameters	0.125	0.115	0.136	0.128	0.121	0.137	change in transmission characterists of the virus
57	dps	doses per seroconversion	2	1.84	2.17	2.07	2.01	2.14	number of vaccine doses required to provide seroconversion
58	abs	age-related testing	1	0.92	1.09	0.91	0.84	0.99	proportion of the population eligible for testing
59	iss	self-isolation	1	0.92	1.09	1.40	1.31	1.48	proportion of symptomatic cases self-isolating
60	rut	Sensitivity to contact rate	1	0.92	1.09	0.83	0.77	0.89	the sensitivity of the contact rate
61	msk	Sensitivity to face coverings	0.04	0.022	0.073	0.023	0.015	0.035	the effect of the use of face coverings on the transmission strength of the virus

Parameters of the COVID-19 DCM Legend: This table lists the acronym of each parameter, along with the prior and posterior densities. If a parameter is not shared by all age groups, the densities for the first age group are shown. These (lognormal)[i] densities are summarised in terms of their expected value and 90% credible intervals. A brief description of each parameter is also provided.

[i] A lognormal distribution precludes negative values for a parameter. Practically, this means one estimates the logarithm of a parameter, under the assumption the log transformed parameter has a normal or Gaussian distribution.
